# Near-Zero Thermal Expansion and Phase Transitions in HfMg_1−*x*_Zn_*x*_Mo_3_O_12_

**DOI:** 10.3389/fchem.2018.00115

**Published:** 2018-04-17

**Authors:** Sailei Li, Xianghong Ge, Huanli Yuan, Dongxia Chen, Juan Guo, Ruofan Shen, Mingju Chao, Erjun Liang

**Affiliations:** ^1^School of Physical Science & Engineering and Key Laboratory of Materials Physics of Ministry of Education, Zhengzhou University, Zhengzhou, China; ^2^College of Science, Zhongyuan University of Technology, Zhengzhou, China; ^3^Department of Physics and Electronic Engineering, Zhoukou Normal University, Zhoukou, China

**Keywords:** thermal expansion, near-zero thermal expansion, phase transition, X-ray diffraction (XRD), Raman spectrum

## Abstract

The effects of Zn^2+^ incorporation on the phase formation, thermal expansion, phase transition, and vibrational properties of HfMg_1−*x*_Zn_*x*_Mo_3_O_12_ are investigated by XRD, dilatometry, and Raman spectroscopy. The results show that (i) single phase formation is only possible for *x* ≤ 0.5, otherwise, additional phases of HfMo_2_O_8_ and ZnMoO_4_ appear; (ii) The phase transition temperature from monoclinic to orthorhombic structure of the single phase HfMg_1−*x*_Zn_*x*_Mo_3_O_12_ can be well-tailored, which increases with the content of Zn^2+^; (iii) The incorporation of Zn^2+^ leads to an pronounced reduction in the positive expansion of the *b*-axis and an enhanced negative thermal expansion (NTE) in the *c*-axes, leading to a near-zero thermal expansion (ZTE) property with lower anisotropy over a wide temperature range; (iv) Replacement of Mg^2+^ by Zn^2+^ weakens the Mo–O bonds as revealed by obvious red shifts of all the Mo–O stretching modes with increasing the content of Zn^2+^ and improves the sintering performance of the samples which is observed by SEM. The mechanisms of the negative and near-ZTE are discussed.

## Introduction

Large difference in coefficients of thermal expansion (CTE) of materials could lead to performance deterioration and even failure of devices due to thermal stress when temperature changes abruptly or frequently. Since most materials expand on heating and contract on cooling, materials with opposite property, namely negative thermal expansion (NTE), are particularly desired for tailoring CTEs. The rediscovery of NTE in ZrW_2_O_8_ in a wide temperature range (Evans et al., 1996, 1997a) triggered continuous efforts on understanding the NTE phenomenon and searching for more NTE materials (Yang et al., 2007; Chen et al., 2013, 2015; Tallentire et al., 2013; Lama et al., 2014; Liu et al., 2014; Peng et al., 2014; Xiao et al., 2014; Hu et al., 2015). To date, different families of NTE materials based on various mechanisms, such as the phonon effect (Pryde et al., 1996; Wang et al., 2011; Bridges et al., 2014; Cheng et al., 2016a; Ge et al., 2016a), magnetovolume effect (Takenaka and Takagi, 2005; Qu et al., 2012; Yan et al., 2014), spontaneous ferroelectric polarization (Chen et al., 2013; Peng et al., 2016), and charge transfer (Long et al., 2009; Azuma et al., 2011; Yamada et al., 2016) have been reported. Among the materials, the family of A_2_M_3_O_12_ (A = transition metal or a mixture of tetravalent and bivalent cations, M = W, Mo) have been particularly attractive, because whose NTEs go over a wide temperature range and can be tuned from low positive to large negative values due to chemical flexibility (Evans et al., 1997b; Suzuki and Omote, 2006; Wu et al., 2009, 2012, 2014; Li et al., 2011; Das et al., 2013; Miller et al., 2013; Song et al., 2014a; Liu et al., 2015; Chen et al., 2016; Cheng et al., 2016a).

In recent years, a number of novel NTE materials have been designed based on the basic structure of A_2_M_3_O_12_ family, including those with a general formula ABM_3_O_12_ where A is tetravalent Hf^4+^ or Zr^4+^ and B is bivalent cation Mg^2+^ or Mn^2+^, and M is W or Mo or a combination of them (Suzuki and Omote, 2004; Baiz et al., 2008; Gindhart et al., 2008; Marinkovic et al., 2008; Song et al., 2013; Li et al., 2014, 2016, 2017; Ge et al., 2016a; Liu et al., 2018) and those with a formula ABM_2_XO_12_ where A and M are the same as in ABM_3_O_12_, B is a trivalent cation and X is P^5+^ or V^5+^ (Chen et al., 2016; Cheng et al., 2016b, 2017; Ge et al., 2016b,c). The most distinct characteristics of the materials with formula ABM_2_XO_12_ are that they exhibit NTE over a wide temperature range and intense photoluminescence in the visible range. Nearly an order higher ionic conductivity was observed for HfMgW_3_O_12_ with respect to the family A_2_M_3_O_12_ (Omote et al., 2011). HfMgMo_3_O_12_ with a linear CTE of 1.02 × 10^−6^ K^−1^ from 298 to 1013 K was reported by Marinkovic et al. (2008). It crystallizes in orthorhombic symmetry with space group *Pnma*(62) or *Pna*2_1_ (33) and transforms to monoclinic structure at 175 K (Miller et al., 2012).

In this paper, we investigate the effects of Zn^2+^ incorporation on the structure, phase transition, thermal expansion, and vibrational properties of HfMgMo_3_O_12_. It is shown that single phase solid solution of HfMg_1−*x*_Zn_*x*_Mo_3_O_12_ can be achieved only for the compositions of *x* ≤ 0.5, otherwise, additional phases of HfMo_2_O_8_ and ZnMoO_4_ appear. The monoclinic to orthorhombic phase transition temperature increases with the content of Zn^2+^ for *x* ≤ 0.5 so that HfMg_0.5_Zn_0.5_Mo_3_O_12_ crystallizes in monoclinic phase and all other samples (*x* ≤ 0.4) adopt orthorhombic structure at room temperature (RT). The incorporation of Zn^2+^ alters the axial CTE differently for each axis and finally results in near-zero thermal expansion (ZTE) property over wide temperature ranges with smaller thermal expansion anisotropy with respect to HfMgMo_3_O_12_. The mechanisms of Zn^2+^ incorporation on the phase transition, thermal expansion and vibrational properties are discussed.

## Experimental

Analytic grade reagents of HfO_2_, MgO, ZnO, and MoO_3_ were mixed with stoichiometric ratios for HfMg_1−*x*_Zn_*x*_Mo_3_O_12_ with *x* = 0.0, 0.1, 0.2, 0.3, 0.4, 0.5, 0.6, 0.7, 0.8, and 1.0. The mixtures were ground in an agate mortar for 2 h, then, pressed under 325 MPa into cylinders with diameter of 10 mm and height of 6 mm using a uniaxial tablet machine. The cylinders were sintered at 1,073 K for 5 h in a muffle furnace in air and cooled down to 300 K naturally.

The as-prepared samples were analyzed by XRD with a PANalytical X'Pert PRO X-ray Diffractometer to identify the crystalline phase. Variable-temperature X-ray powder data were collected on a Rigaku (Japan, SmartLab 3KW) diffractometer with Cu Kα (λ = 0.15405 nm) radiation. Diffraction data were collected with a step size of 0.01° in the 2θ range of 10°–120°. The sample was heated at a rate of 10 K/min and remained at each measurement temperature for 5 min before measurement. Unit cell dimensions above the phase transition temperature were determined with software of PowderX. Variable-temperature/RT Raman spectra were recorded with A LabRAM HR Evolution Raman spectrometer (France HORIBA JobinYvon S.A.A.) equipped with a Linkam THMS600 Heating and Freezing Stage (Japan Hightech) (an accuracy of ±0.1 K). The excitation wavelength is 633 nm and low excitation laser power is necessary to avoid local heating by the laser. The microstructures and energy dispersive spectra of the samples were examined with a scanning electron microscope (SEM, Model Quanta 250). The relative length changes were measured with LINSEIS DIL L75 dilatometer at the heating and cooling rates of 5 K/min.

## Results and discussion

**Figure 1:**
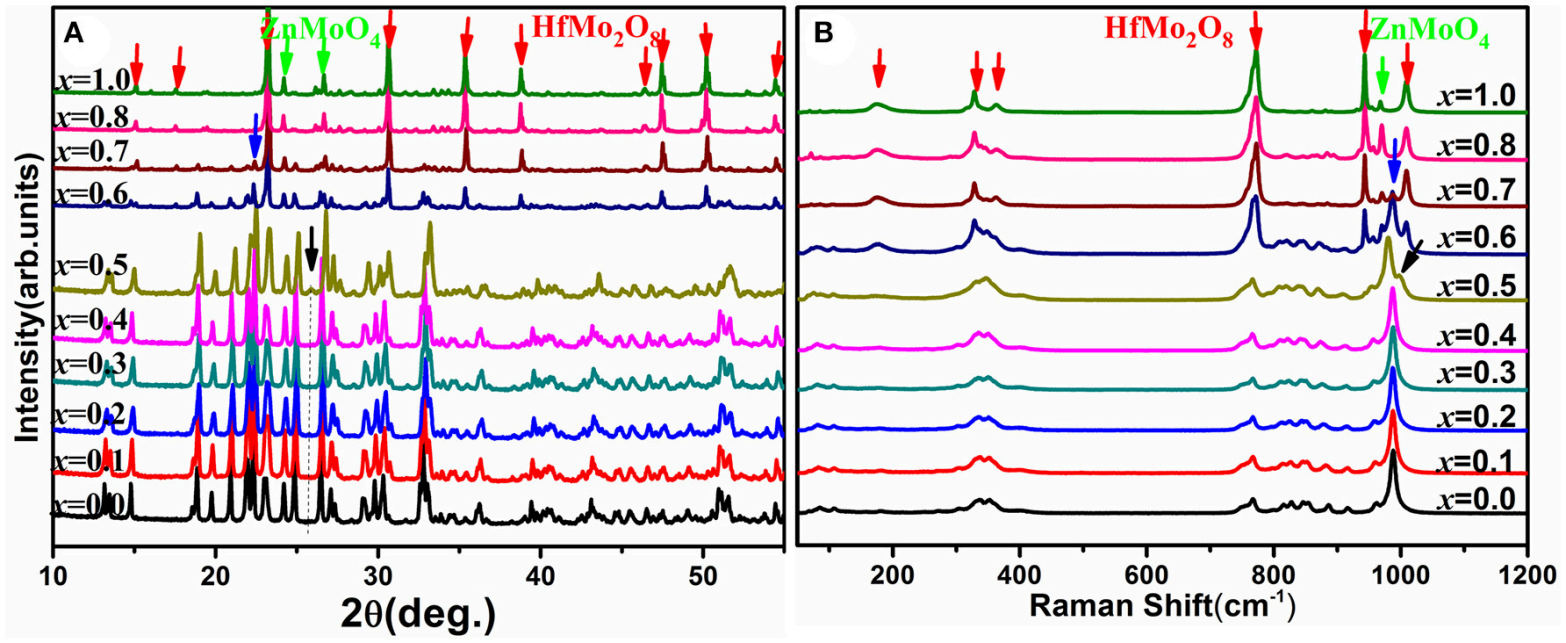
**(A)** X-ray diffraction patterns of the solid solutions of HfMg_1−*x*_Zn_*x*_Mo_3_O_12_; **(B)** Raman spectra of the solid solutions of HfMg_1−*x*_Zn_*x*_Mo_3_O_12_.

Figure 1A shows the XRD patterns of the solid solutions of HfMg_1−*x*_Zn_*x*_Mo_3_O_12_. When *x* = 0.0, the diffraction peaks are corresponding to HfMgMo_3_O_12_, which adopts an orthorhombic structure with space group *Pnma* or *Pna*2_1_ (Marinkovic et al., 2008). No obvious changes in the XRD patterns could be observed with increasing the content of Zn^2+^ till *x* = 0.4. It is reasonable to conclude that HfMg_1−*x*_Zn_*x*_Mo_3_O_12_ for *x* ≤ 0.4 crystallized in an orthorhombic structure. Nevertheless, some subtle changes are observed for *x* = 0.5, such as the weak peak appearing at about 25.6° which is characteristic for a monoclinic structure (Song et al., 2014b; Ge et al., 2016a) of ABMo_3_O_12_. HfMg_0.5_Zn_0.5_Mo_3_O_12_ at RT is thus identified as a monoclinic structure. The XRD patterns change obviously with further increasing content of Zn^2+^. Detailed analyses show that the newly appeared peaks correspond well to HfMo_2_O_8_ and ZnMoO_4_ (Reichelt et al., 2000; Allen et al., 2004), respectively.

The above analysis is supported by Raman spectroscopic analysis (Figure 1B). The Raman spectra are consistent with each other for *x* ≤ 0.4 while the Raman band at 988 cm^−1^ splits into two bands at 980 and 998 cm^−1^ for *x* = 0.5 (as indicated by the black arrowhead), which is characteristic for a phase transition from higher orthorhombic symmetry to lower monoclinic symmetry (Li et al., 2011; Ge et al., 2016a) for the ABMo_3_O_12_ family. Distinct changes of the Raman spectra occur for higher content of Zn^2+^. The new Raman bands at about 175, 328, 362, 772, 943, and 1,008 cm^−1^ correspond well to HfMo_2_O_8_ (Liang et al., 2008b) and that around 968 cm^−1^ arises from ZnMoO_4_ (Ahsaine et al., 2016). Both XRD and Raman analyses demonstrate that a single phase solid solution of HfMg_1−*x*_Zn_*x*_Mo_3_O_12_ is only possible for *x* ≤ 0.5 and additional phases of HfMo_2_O_8_ and ZnMoO_4_ form for *x* ≥ 0.6. At RT, HfMg_1−*x*_Zn_*x*_Mo_3_O_12_ for *x* ≤ 0.4 adopt an orthorhombic structure while HfMg_0.5_Zn_0.5_Mo_3_O_12_ crystallizes in a monoclinic structure.

**Figure 2:**
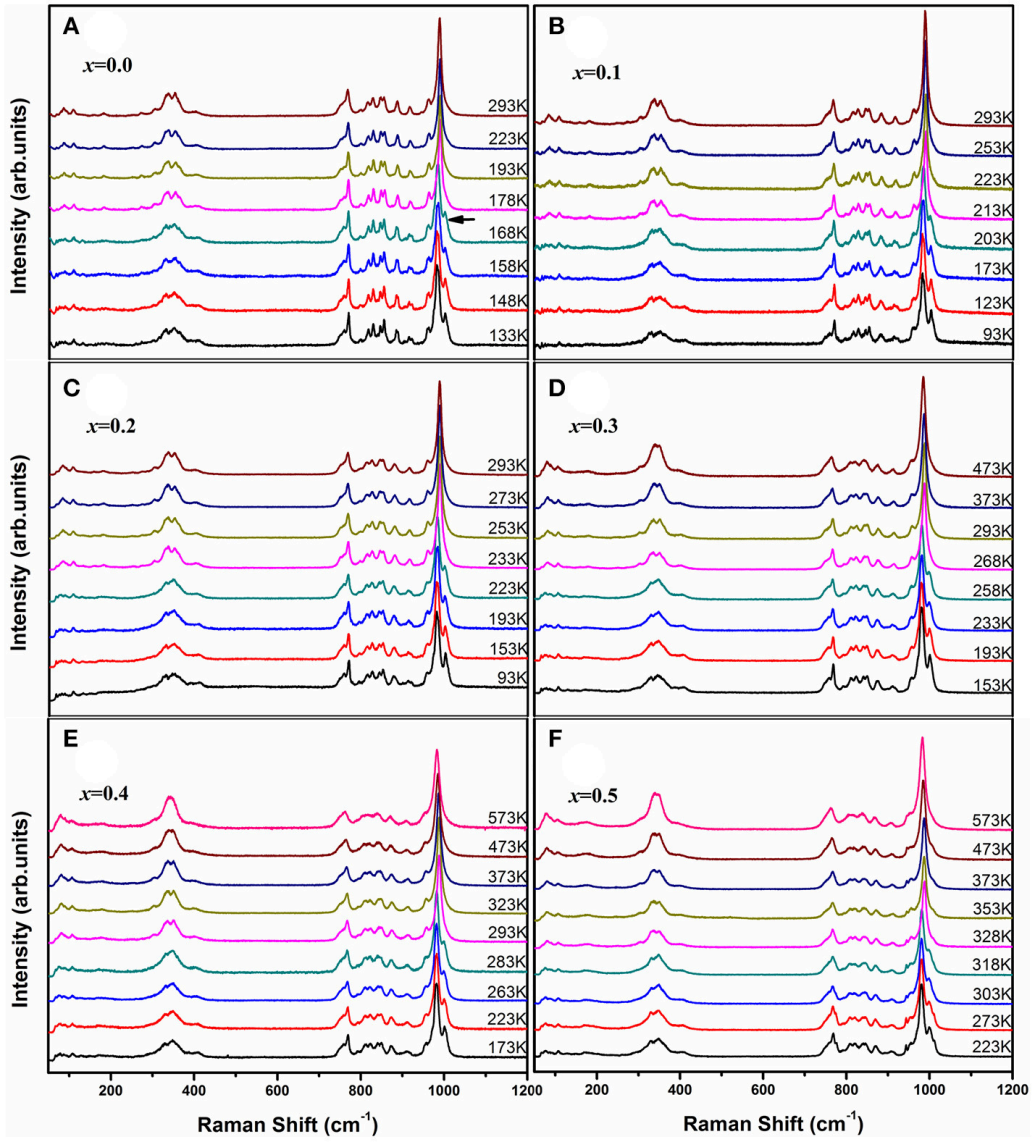
Temperature-dependent Raman spectra of HfMg_1−*x*_Zn_*x*_Mo_3_O_12_ with *x* = 0.0 **(A)**, 0.1 **(B)**, 0.2 **(C)**, 0.3 **(D)**, 0.4 **(E)**, and 0.5 **(F)**.

Raman spectroscopy is very sensitive to the monoclinic-to-orthorhombic phase transition (Li et al., 2011, 2016; Ge et al., 2016a). In order to get some insights into the influence of Zn^2+^ on the phase transition, we carried out temperature-dependent Raman spectral observation of HfMg_1−*x*_Zn_*x*_Mo_3_O_12_ (*x* ≤ 0.5) as shown in Figure 2. The XRD analyses suggest that HfMg_1−*x*_Zn_*x*_Mo_3_O_12_ (*x* ≤ 0.5) have similar open framework structure as HfMgMo_3_O_12_. In the orthorhombic phase, there are four molecular formulas in a unit cell, in which each MoO_4_ tetrahedron sharing its four vortexes with HfO_6_/MgO_6_ octahedra and each HfO_6_/MgO_6_ octahedron shares its corners with six MoO_4_ tetrahedra. Hf and Mg are alternatively aligned in the [010] direction forming a quasi-layered structure (Omote et al., 2011). The Raman modes from 1,050 to 900 cm^−1^, from 900 to 750 cm^−1^, from 400 to 320 cm^−1^, and from 320 to 280 cm^−1^ are identified as symmetric stretching (ν_1_), asymmetric stretching (ν_3_), asymmetric bending (ν_4_), and symmetric bending (ν_2_) modes in the MoO_4_ tetrahedra, respectively (Liang et al., 2008a; Li et al., 2011). Figure 2A shows the temperature dependent Raman spectra of HfMgMo_3_O_12_. The most distinctive change of the Raman spectra is the disappearance of the band at about 1,001 cm^−1^ with temperature increase from 168 to 178 K, which can be regarded as characteristic of the phase transition from low temperature monoclinic to high temperature orthorhombic structure (Li et al., 2011; Ge et al., 2016a). The phase transition temperature agrees well with the result derived from XRD analysis (Miller et al., 2012). In Figures 2B–F we present the temperature dependent Raman spectra for Zn^2+^-containing samples. It is shown that the vanishing of the characteristic Raman band for the monoclinic structure occurs in the ranges of 168–178, 203–213, 223–233, 258–268, 283–293, and 318–328 K for *x* = 0.0, 0.1, 0.2, 0.3, 0.4, and 0.5, respectively, demonstrating that the phase transition temperature increases with the content of Zn^2+^.

In the orthorhombic structure of HfMg_1−*x*_Zn_*x*_Mo_3_O_12_ (*x* ≤ 0.5), the four vortexes of each MoO_4_ tetrahedron are shared with two HfO_6_ and two MgO_6_/ZnO_6_ octahedra while each HfO_6_/MgO_6_/ZnO_6_ octahedron shares its corners with six MoO_4_ tetrahedra. Statistically, each MoO_4_ tetrahedron links to 0.0, 0.2, 0.4, 0.8, and 1.0 ZnO_6_ octahedron for *x* = 0.0, 0.1, 0.2, 0.3, 0.4, and 0.5. Since the ionic radius of Zn^2+^ is 74 pm which is slightly larger than that of Mg^2+^ (72 pm), large lattice distortion and increase in phase transition temperature is not expected if only the ionic radius is considered. The experimentally observed obvious increase in phase transition temperature is therefore attributed to the difference in electronegativity between Zn^2+^ (1.65 Pauling) and Mg^2+^ (1.31 Pauling). Replacement of Mg^2+^ by Zn^2+^ causes an increase in electronegativity at theZn^2+^-cation side and a decrease in the effective negative charge on oxygen, and hence a decrease in the oxygen-oxygen repulsion. With increasing the content of Zn^2+^, oxygen-oxygen attractive forces increase, causing the network collapse transition to occur at higher temperatures (Evans et al., 1997b).

**Figure 3:**
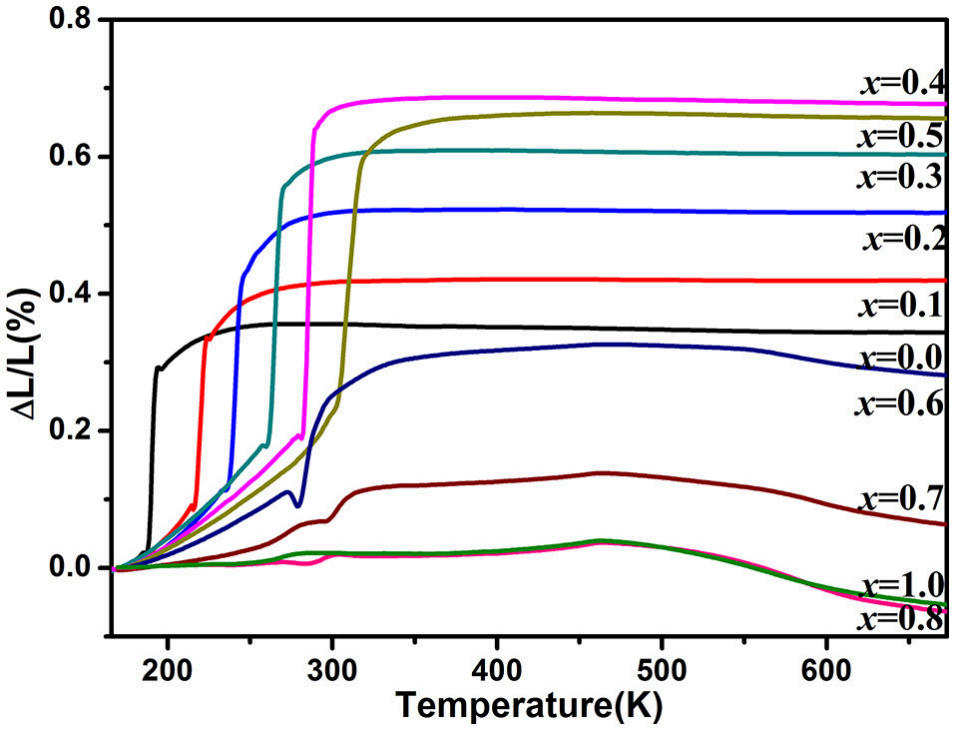
Variations of relative length of the solid solutions of HfMg_1−*x*_Zn_*x*_Mo_3_O_12_ with increasing temperature from 170 to 673 K.

Figure 3 shows the relative length changes of sintered cylinders with increasing temperature measured by dilatometry. All the samples for *x* ≤ 0.6 exhibit abrupt length increase around the temperature of monoclinic to orthorhombic phase transition. The phase transition temperature increases with increasing the content of Zn^2+^ except the one for *x* = 0.6 whose phase transition temperature is lower than that of *x* ≤ 0.5 due to the generation of HfMo_2_O_8_ and ZnMoO_4_. In this case, it can be deduced that the real content of Zn^2+^ in HfMg_1−*x*_Zn_*x*_Mo_3_O_12_ is lower than HfMg_0.5_Zn_0.5_Mo_3_O_12_. These results comply well with the above Raman spectroscopic analyses. The CTEs are calculated from the relative length change and shown in the Table 1. It indicates that all the single phase samples present excellent near-ZTE property above the phase transition temperature. It is interesting to notice that even for the multi-phase samples for *x* = 0.8 and 1.0, a near-ZTE property in a wide temperature range are realized. However, in this paper we focus on the effect of Zn^2+^ incorporation on the structure and properties of the single phase HfMg_1−*x*_Zn_*x*_Mo_3_O_12_.

**Table 1:** Values of CTEs of HfMg_1−*x*_Zn_*x*_Mo_3_O_12_.

**Samples (*x*)**	**CTEs (10^−6^ K^−1^)**	**Fit range (K)**
0.0	−0.21	248–673
0.1	0.05	303–673
0.2	−0.05	308–673
0.3	−0.09	323–673
0.4	−0.11	343–673
0.5	−0.11	373–673

**Figure 4:**
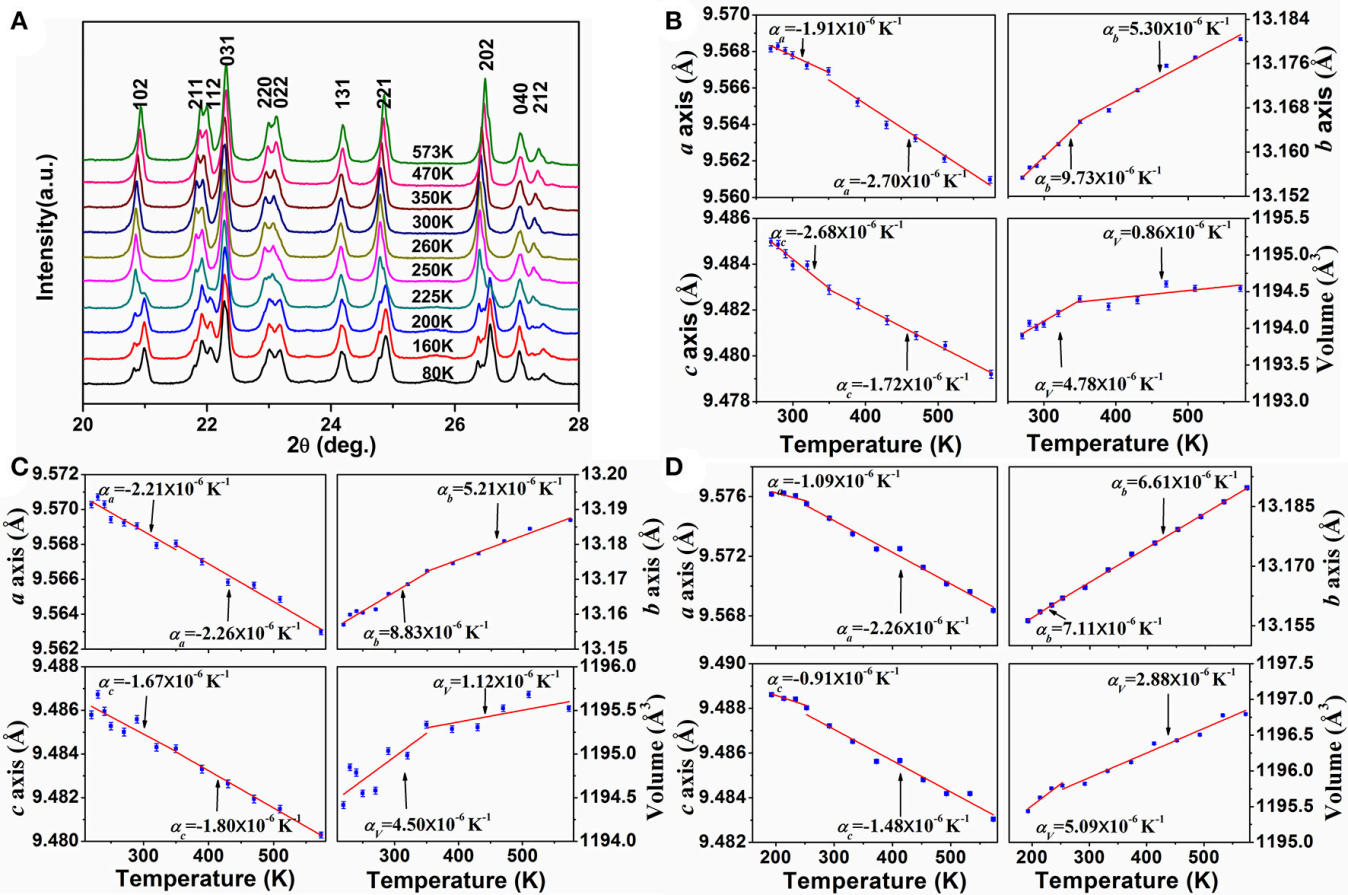
**(A)** Variable-temperature XRD patterns of HfMg_0.7_Zn_0.3_Mo_3_O_12_ (The XRD patterns were measured for 2θ = 10°-120°, here only a small range is shown for clarity); Changes of lattice constants and volume of HfMg_1−*x*_Zn_*x*_Mo_3_O_12_ with temperature: **(B)** for *x* = 0.3, **(C)** for *x* = 0.2, **(D)** for HfMgMo_3_O_12_.

In order to get insight into the axial thermal expansion property, we carried out temperature-dependent powder XRD measurements for the samples of *x* = 0.2 and 0.3. For comparison, variable-temperature XRD data of HfMgMo_3_O_12_ were also collected. Figure 4A shows the selected temperature-dependent XRD patterns for HfMg_0.7_Zn_0.3_Mo_3_O_12_ at different temperatures. It is obvious that its XRD pattern changes distinctively around 225 K, which is attributed to the phase transformation from lower temperature monoclinic to higher temperature orthorhombic structure. Lattice constants and cell volume at each temperature are calculated and given in Figure 4B. It is evident that the *a*- and *c*-axes contract while the *b*-axis expands with increasing temperature. The CTEs for the *a*-, *b*-, and *c*-axes and volume are calculated to be $\alpha_{a}\;=\;-2.70\;\times \;10^{-6}\;K^{-1}$, $\alpha_{b}\;=\;5.30\;\times \;10^{-6}\;K^{-1}$, $\alpha_{c}\;=\;-1.72\;\times \;10^{-6}\;K^{-1}$, $\alpha_{V}=0.86\;\times \;10^{-6}\;K^{-1}\left(350-573\;K\right)$, respectively. This gives rise to a linear CTE $\alpha_{l}\;=\;0.29\;\times \;10^{-6}\;K^{-1}$. Similar axial thermal expansion behaviors are obtained for HfMg_0.8_Zn_0.2_Mo_3_O_12_ from temperature dependent XRD measurements (not shown here). The changes of its lattice constants and volume with temperature are given in Figure 4C. The CTEs for the *a*-, *b*-, and *c*-axes and volume are calculated to be $\alpha_{a}\;=\;-2.26\;\times \;10^{-6}\;K^{-1}$, $\alpha_{b}\;=\;5.21\;\times \;10^{-6}\;K^{-1}$, $\alpha_{c\;}=\;-1.80\;\times \;10^{-6}\;K^{-1}$, $\alpha_{V}\;=\;1.12\;\times \;10^{-6}\;K^{-1}$, respectively, corresponding to a linear CTE $\alpha_{l}\;=\;0.37\;\times \;10^{-6}\;K^{-1}\;\left(350-573\;K\right)$. These results are consistent with the values measured by dilatometry, confirming HfMg_0.7_Zn_0.3_Mo_3_O_12_ and HfMg_0.8_Zn_0.2_Mo_3_O_12_ being intrinsically ZTE materials. Figure 4D shows the changes of lattice constants and volume of HfMgMo_3_O_12_ with temperature.

Considering the fact that the intrinsic linear CTE of HfMgMo_3_O_12_ is 1.02 × 10^−6^ *K*^−1^ (Marinkovic et al., 2008), it is reasonable to conclude that the incorporation of Zn^2+^ reduces the linear CTE and results in near ZTE of HfMg_1−*x*_Zn_*x*_Mo_3_O_12_. A comparison of the axial CTEs for HfMgMo_3_O_12_, HfMg_0.8_Zn_0.2_Mo_3_O_12_, and HfMg_0.7_Zn_0.3_Mo_3_O_12_ are given in Table 2. It is found that partial substitution of Mg^2+^ by Zn^2+^ leads to a significant reduction of the CTE in the *b*-axis and an increase of the NTE in the *c*-axis, resulting in a near ZTE and lower anisotropy of thermal expansion in the Zn-containing compounds with respect to HfMgMo_3_O_12_ (see Table 2). The anisotropy of thermal expansion is defined as the maximum difference in the axial thermal expansion coefficients (Srikanth et al., 1992; Miller et al., 2013). The near zero linear thermal expansion and lower anisotropy property of the Zn-containing compounds suggest that they could withstand higher thermal shock resistance.

**Table 2:** Intrinsic thermal expansion coefficients (α) for HfMg_0.7_Zn_0.3_Mo_3_O_12_ and HfMg_0.8_Zn_0.2_Mo_3_O_12_ as obtained from variable-temperature XRD and literature and experimental values for HfMgMo_3_O_12_.

**Sample (Structure)**	**Fit range (K)**	**α_*a*_ (10^−6^ K^−1^)**	**α_*b*_ (10^−6^ K^−1^)**	**α_*c*_ (10^−6^ K^−1^)**	**α_*l*_ (10^−6^ K^−1^)**	**Δα_*max*_ (10^−6^ K^−1^)**	**References**
HfMgMo_3_O_12_ (Orthorhombic)	298–1,013	−3.44	8.0	−1.49	1.02	11.44	Marinkovic et al., 2008
HfMgMo_3_O_12_ (Orthorhombic)	253–573	−2.26	6.61	−1.48	0.96	8.87	This work
HfMg_0.8_Zn_0.2_Mo_3_O_12_ (Orthorhombic)	350–573	−2.26	5.21	−1.80	0.37	7.47	This work
HfMg_0.7_Zn_0.3_Mo_3_O_12_ (Orthorhombic)	350–573	−2.70	5.30	−1.72	0.29	8	This work

The difference in the linear CTEs measured by XRD and dilatometry could be understood by the microstructural effects. In contrast to XRD measurement which gives the thermal expansion property of cell lattice, dilatometry reveals the bulk thermal expansion property, including both intrinsic (thermal expansion of a material arising from the lattice dynamics) and extrinsic (thermal expansion related to microstructures such as texture, grain size, grain boundaries, poses, and microcracks) effects. The difference measured by the two methods reflects the extrinsic effect in the sintered bulk, which, on heating, can add a small negative component to the intrinsic linear expansion coefficients. Generally speaking, a smaller difference suggests a better sintered quality of the bulk material which is desired for most applications. The absolute differences for the Zn-containing compounds (Δ$\alpha_{l}\;=\;0.42\;\times \;10^{-6}\;K^{-1}$ for *x* = 0.2 and Δ$\alpha_{l}\;=\;0.38\;\times \;10^{-6}\;K^{-1}$ for *x* = 0.3) are obviously smaller than that for HfMgMo_3_O_12_ (Δ$\alpha_{l\;}=\;1.17\;\times \;10^{-6}\;K^{-1}$). It means that partial substitution of Mg^2+^by Zn^2+^ in HfMgMo_3_O_12_ could improve the sintering performance of the material and minimize the possible contributions of extrinsic effects. The analysis is supported by microstructural observation.

**Figure 5:**
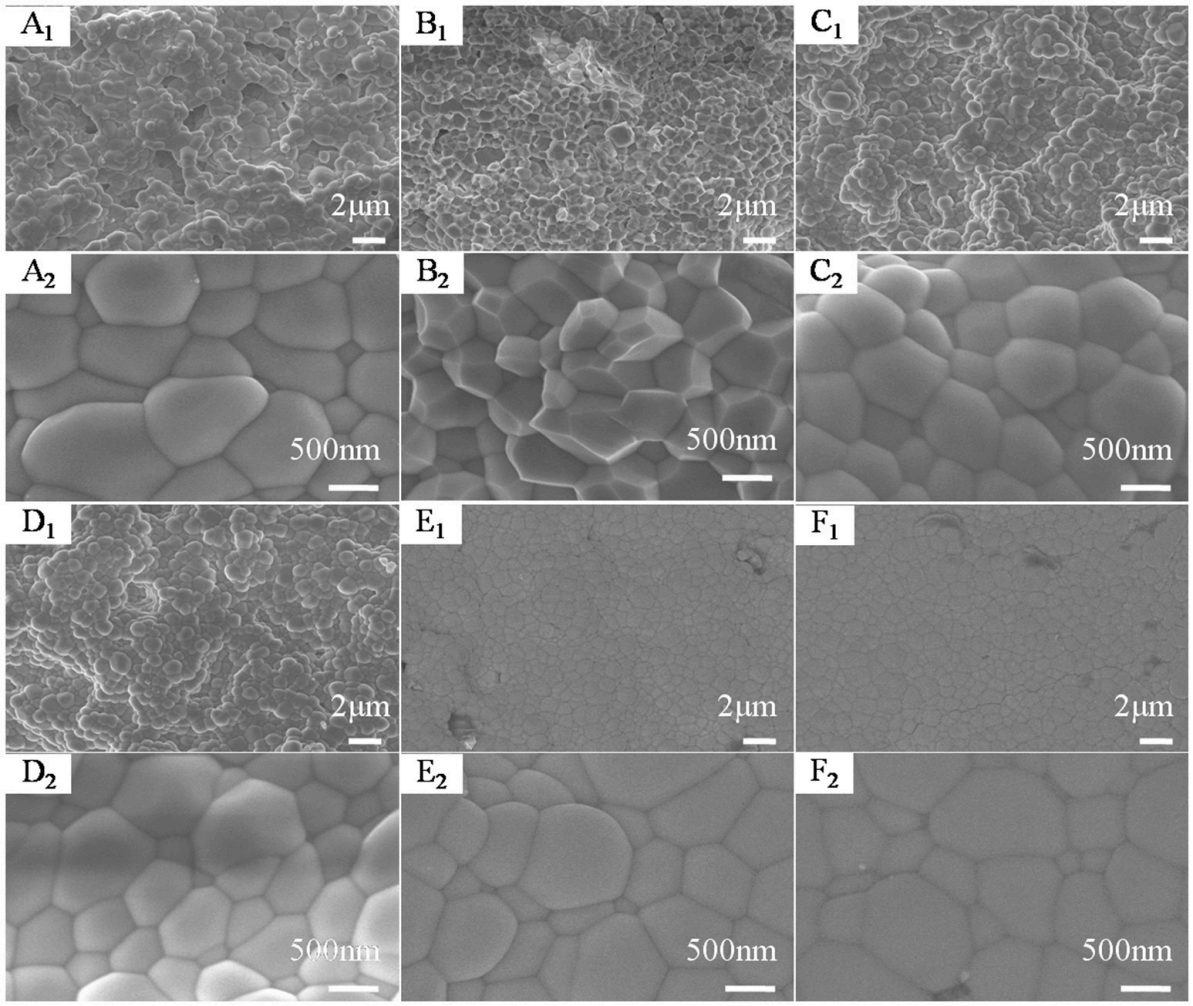
SEM images of HfMg_1−*x*_Zn_*x*_Mo_3_O_12_ with *x* = 0.0 **(A)**, 0.1 **(B)**, 0.2 **(C)**, 0.3 **(D)**, 0.4 **(E)**, and 0.5 **(F)**.

Figure 5 shows the SEM images of HfMg_1−*x*_Zn_*x*_Mo_3_O_12_ ceramics with *x* = 0.0 (Figure 5A), 0.1 (Figure 5B), 0.2 (Figure 5C), 0.3 (Figure 5D), 0.4 (Figure 5E), and 0.5 (Figure 5F). The micro morphology of the sample for *x* = 0.1 is dominated by well crystallized polyhdra or truncated polyhedra and the average particle size is obviously smaller than that of HfMgMo_3_O_12_. With increasing the content of Zn^2+^, the polyhedra become more rounded. Compared to HfMgMo_3_O_12_, the incorporation of Zn^2+^ seems to lead to less pores in the ceramic bodies and pores can hardly be found in the solid solutions for *x* = 0.1–0.3. It illustrates that proper amount incorporation of Zn^2+^ favors the formation of uniform distribution of particles and efficient reduction of porosity in the sintered body.

**Figure 6:**
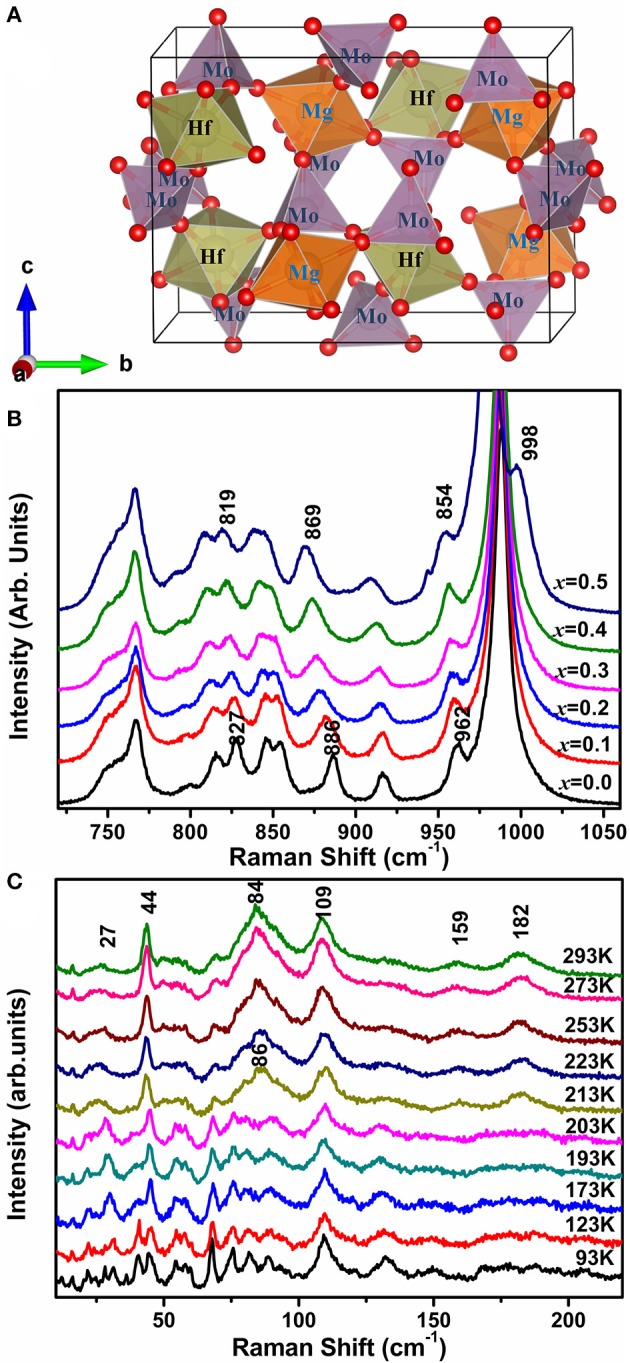
**(A)** Schematic diagram of HfMgMo_3_O_12_ building block (red sphere indicates oxygen atom); **(B)** Raman spectra of HfMg_1−*x*_Zn_*x*_Mo_3_O_12_ in the high wavenumber regions; **(C)** Temperature-dependent Raman spectra of HfMg_0.9_Zn_0.1_Mo_3_O_12_ in the low wavenumber region.

The schematic diagram of HfMgMo_3_O_12_ is given in Figure 6A to help us understand the mechanism of the phenomenons. In HfMg_1−*x*_Zn_*x*_Mo_3_O_12_ (x ≤ 0.5), Zn^2+^ is expected to substitute for Mg^2+^ due to the same valence and similar cation radius and each ZnO_6_ octahedron shares all its corners with six MoO_4_ tetrahedra. In order to see the bond strength changes induced by local electronic environment upon substitution of Zn^2+^for Mg^2+^, we show in Figure 6B the Raman spectra of the stretching region for *x* = 0.0, 0.1, 0.2, 0.3, 0.4, and 0.5. It is obvious that all the stretching modes shift successively to lower wavenumbers with increasing the content of Zn^2+^, indicating a softening of the Mo-O bonds upon incorporation of Zn^2+^. Once an Mg^2+^ is replaced by Zn^2+^, the local electronic equilibrium around the MoO_4_ tetrahedron is broken. Zn^2+^ has obviously a higher electronegativity and ability to drag electrons to the ZnO_6_ octahedron from its connected six MoO_4_ tetrahedra than Mg^2+^, resulting in the weakening of the Mo–O bonds. The differences in ionic radius and electronegativity could cause a slight rotation of the connected polyhedra and hence the M-O-M linkages. This is probably the reason that the positive expansion of the *b*-axis is pronouncedly reduced and the NTE in the *c*-axes become more negative, resulting hence in a lower anisotropy in thermal expansion and near-zero CTEs of the Zn-containing compounds. Due to the large difference in electronegativity between Zn^2+^ (1.65 Pauling) and Mg^2+^ (1.31 Pauling), the more of Zn^2+^ is incorporated, the more of the MoO_4_ tetrahedra get distorted as revealed by Raman spectroscopy, resulting in larger distortion and instability of the lattice. When the forming energy for the single phase exceeds that for the multi-phases, then the multi-phases form.

Figure 6C shows the temperature-dependent Raman spectra of HfMg_0.9_Zn_0.1_Mo_3_O_12_ in the low wavenumber region. Obvious change of the Raman spectra occur between 203 and 213 K, corresponding to the monoclinic to orthorhombic phase transition, such as the appearance of new Raman modes at about 27,44, 86, 159, and 182 cm^–1^. The modes at about 44 and 86 cm^–1^ are split into two or three modes in the low temperature phase and become degenerated in the high temperature phase. The low wavenumber modes arise from the external librational and translational vibrations of the connected octahedra–tetrahedra, or the librational and translational motions of metal ions in the Hf(Mg/Zn)–O–Mo linkages, which can also be regarded as the transverse vibrations of the bridging oxygen from the point of view of relative movement. Such an harmonic vibrations along with the distortion of the polyhedra are believed to be the origin of the NTE in the open frame work structure since they bring the two end atoms closer upon heating (Evans, 1999; Ding et al., 2008; Marinkovic et al., 2009; Wang et al., 2013).

## Conclusion

Solid solutions of HfMg_1−*x*_Zn_*x*_Mo_3_O_12_ with near-ZTE are successfully synthesized by solid state reaction and the effects of Zn^2+^ incorporation on the phase formation, thermal expansion, phase transition, and vibrational properties and micro-morphologies are investigated by XRD, dilatometry, Raman spectroscopy, and SEM. It is shown that (i) single phase formation is only possible for *x* ≤ 0.5, otherwise, additional phases of HfMo_2_O_8_ and ZnMoO_4_ generate; (ii) HfMg_1−*x*_Zn_*x*_Mo_3_O_12_ crystallize in an orthorhombic structure for *x* ≤ 0.4 and in a monoclinic structure for *x* = 0.5 at RT; (iii) The phase transition temperature from monoclinic to orthorhombic structure increases with the content of Zn^2+^, which occurs within 168–178, 203–213, 223–233, 258–268, 283–293, and 318–328 K for *x* = 0.0, 0.1, 0.2, 0.3, 0.4, and 0.5, respectively; (iv) The incorporation of Zn^2+^ leads to an pronounced reduction in the positive expansion of the *b*-axis and an enhanced NTE in *c*-axes, making the Zn-containing materials exhibit near-ZTE over a wide temperature range and lower anisotropy in thermal expansion in the orthorhombic phase; (v) Replacement of Mg^2+^ by Zn^2+^ breaks the local electronic equilibrium around the MoO_4_ tetrahedron and weakens the Mo–O bonds, leading to obvious red shifts of all the Mo–O stretching modes with increasing the content of Zn^2+^ due to obviously higher electronegativity of Zn^2+^ than Mg^2+^; (vi) The incorporation of Zn^2+^ improves sintering property of samples, minimizing the possible contributions of extrinsic effects such as pores, which is preferred for most applications.

## Author contributions

EL conceived the idea and supervised the research. SL and RS are in charge of the synthesis and part measurements of the materials. XG, HY, and DC are in charge of the thermal expansion and Raman characterization. JG and MC are in charge of the XRD characterization and structural analyses. SL, XG, and EL are in charge of the manuscript preparation.

### Conflict of interest statement

The authors declare that the research was conducted in the absence of any commercial or financial relationships that could be construed as a potential conflict of interest.
